# A comprehensive multi-evidence framework for network pharmacology-based prediction of dietary flavonoid effects

**DOI:** 10.3389/fnut.2026.1792521

**Published:** 2026-04-14

**Authors:** Koyo Fujisaki, Osei Horikoshi, Yukitoshi Nagahara, Kengo Morohashi

**Affiliations:** 1 Faculty of Science and Technology, Department of Applied Chemistry and Bioscience, Chitose Institute of Science and Technology, Chitose, Hokkaido, Japan; 2 Division of Life Science, School of Science and Engineering, Tokyo Denki University, Hiki-gun, Saitama, Japan

**Keywords:** dietary bioactives, flavonoids, food composition database, multi-evidence assessment, network pharmacology, polypharmacology, precision nutrition

## Abstract

Dietary flavonoids associate with various aspects of disease prevention, yet systematic frameworks integrating computational prediction with experimental and epidemiological evidence remain limited. We develop a multi-tiered network pharmacology framework that quantitatively predicts flavonoid-related therapeutic properties and supports these predictions with integrated computational, experimental, and epidemiological evidence. We constructed a master network of 17,869 human proteins, 14 dietary flavonoids, and 1,496 FDA-approved drugs (278,768 interactions). Flavonoids averaged 45.3 target proteins compared to 16.8 for FDA-approved drugs (2.7-fold higher; *p* = 7.5 × 10^−4^), reflecting multi-target architecture. Statistical analysis using target protein overlap (Fisher's exact test) revealed that 71.4% of flavonoids showed significant associations with cardiovascular drugs and 78.6% with anticancer drugs. Experimental validation in cancer cell models demonstrated high predictive accuracy: flavonoids with strong computational associations to anticancer drugs (luteolin: –log_10_ *p* = 10.5; myricetin: –log_10_ *p* = 9.9) exhibited potent cytotoxicity (LC_50_ ~30 μM), whereas weakly associated flavonoids remained inactive (LC_50_ > 200 μM). Computational association strength explained 84% of the variance in experimental potency (Pearson *r* = 0.918; R^2^ = 0.843), providing quantitative experimental support of network pharmacology predictions for dietary bioactives. Translating predictions to 506 foods yielded 685 food-ATC therapeutic combinations. Systematic PubMed analysis identified literature-supported evidence for 96 associations (132 unique references), achieving 47.1% cardiovascular predictions showing observational consistency with published studies. Food category analysis identified tomato, cranberry, tea, orange, and blueberry products with strongest evidence (18–40 items). This multi-evidence framework enables evidence-based prediction of dietary polypharmacological effects and provides a computational foundation to generate hypotheses for precision nutrition.

## 1 Introduction

Dietary choices fundamentally shape chronic disease risk. Among dietary bioactives, flavonoids—ubiquitous plant polyphenols found in fruits, vegetables, and beverages—have emerged as critical protective agents. Epidemiological evidence documents that flavonoid-rich foods reduce chronic disease risk, particularly cardiovascular disease and malignancy (1–3). Individual flavonoids—luteolin, quercetin, kaempferol, catechin, epicatechin, and naringenin—demonstrate potent cardiovascular protection through improved endothelial function and blood pressure regulation (4). Others including quercetin, kaempferol, myricetin, apigenin, and luteolin demonstrate anticancer activity in preclinical models (5).

Modern drug discovery adheres to Ehrlich's “magic bullet” paradigm, prioritizing selective ligands that target single disease-related proteins with high affinity (6). This “one gene, one drug, one disease” framework presumes selectivity minimizes adverse effects (7), systematically favoring compounds with narrow target profiles. Consequently, the FDA has approved few flavonoid-containing drugs despite strong evidence for their bioactivity, as flavonoids inherently engage multiple protein targets. Network pharmacology, however, challenges this reductionist premise (8).

Biological systems exhibit inherent redundancy where gene deletion frequently produces minimal phenotypic effects (9). Scale-free network architectures resist random perturbations yet require coordinated multi-node modulation for therapeutic efficacy (10, 11). Complex pathologies—particularly polygenic diseases including cancer, cardiovascular disease, and neurodegeneration—accordingly reflect extended protein network perturbations rather than isolated molecular defects (12). Network pharmacology, which integrates drug-protein-disease interactions to identify synergistic multi-target combinations (13), has thus gained prominence as approved drugs increasingly exhibit polypharmacological properties (14, 15). This paradigm shift from single-target selectivity toward network-based strategies (13) has been validated experimentally: Valle et al. (16) demonstrated that network proximity between polyphenol targets and disease proteins predicts therapeutic effects for rosmarinic acid's cardiovascular benefits. While this framework established the potential of network-based polyphenol analysis, systematic validation across diverse flavonoid structures and therapeutic domains remains limited.

Dietary flavonoids provide an ideal system to address this gap and extend this framework. These compounds associate with multiple proteins. Such multi-target engagement, typically problematic for conventional drugs, remains safe when flavonoids are consumed as dietary constituents. This apparent paradox suggests that multi-target engagement is not inherently problematic when perturbations remain within physiologically tolerable ranges. Flavonoid safety likely reflects modest binding affinities and physiological concentrations that produce coordinated network modulation rather than disruptive single-target effects characteristic of high-affinity pharmaceuticals. Moreover, flavonoid-rich foods contain multiple structurally diverse flavonoids, raising the possibility that polypharmacological effects arise from synergistic combinations rather than individual compounds. Despite this epidemiological evidence (1–3), quantitative frameworks that integrate both individual and combinatorial multi-target properties remain lacking.

**Figure 1:**
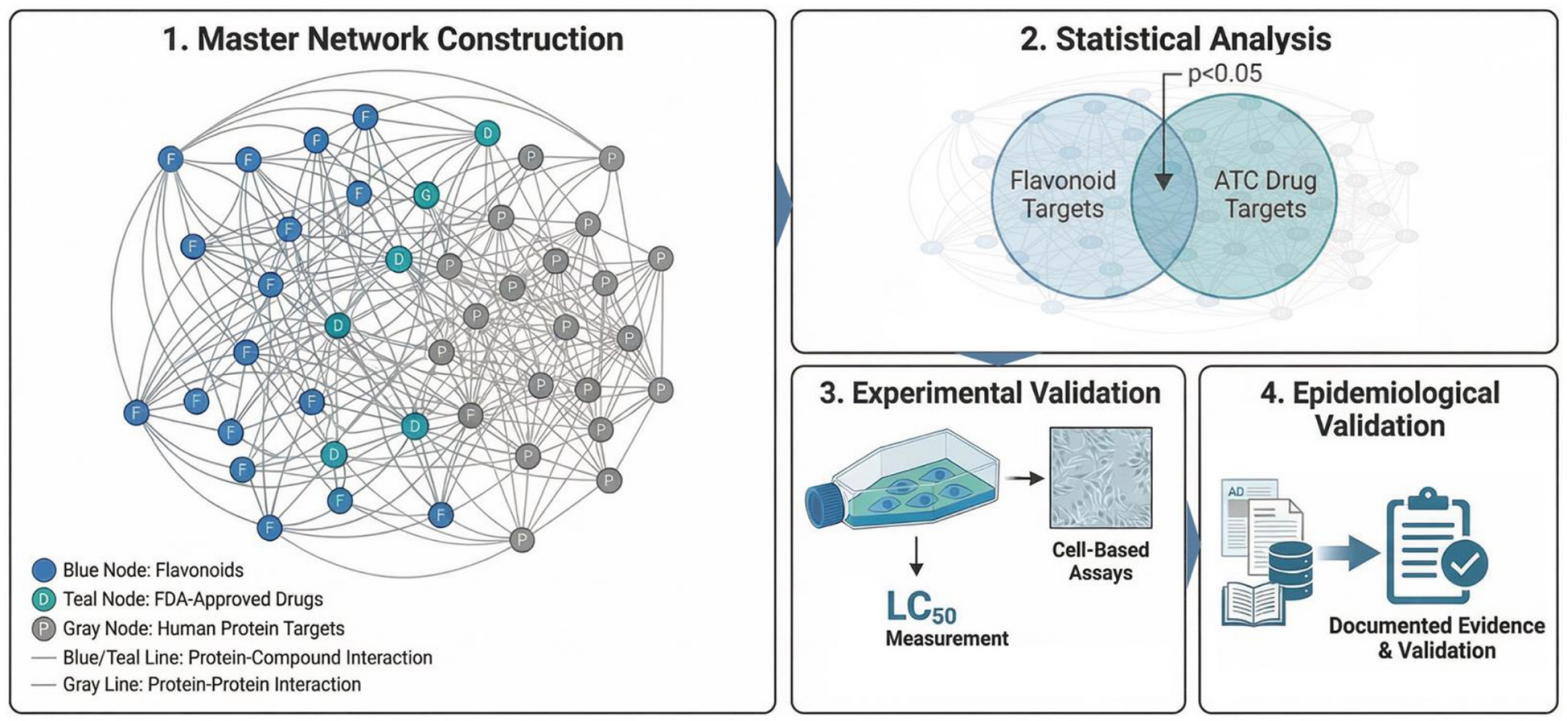
Overview of the multi-tiered framework for dietary flavonoid effect prediction. **(1)** Master network of flavonoid-protein and drug-protein interactions. **(2)** Statistical enrichment analysis of flavonoid target overlap with therapeutic drug categories. **(3)** Cell-based bioassays measuring LC_50_ values. **(4)** Epidemiological assessment via literature survey. Framework integrates network pharmacology, experimental validation, and epidemiological evidence for identifying dietary sources with therapeutic potential.

To address this gap, we hypothesized that dietary flavonoid combinations possess quantifiable pharmacological properties amenable to systematic prediction and experimental evaluation. We developed a comprehensive approach: (1) constructing a master network of protein-protein and protein-compound interactions incorporating flavonoids, FDA-approved drugs, and human protein targets; (2) statistically identifying flavonoids whose target profiles align with therapeutic drug categories; (3) experimentally evaluating computational predictions through cell-based assays; and (4) assessing epidemiological consistency by surveying published literature for documented food-health associations. This multi-evidence framework establishes a systematic approach for identifying dietary sources with therapeutic potential, bridging nutritional science and network pharmacology (Figure 1).

## 2 Materials and methods

### 2.1 Data collection and curation

#### 2.1.1 Protein interaction networks

Human protein-protein interactions were obtained from STRING database version 11.5, which integrates evidence from automated text mining, experimental data, and computational predictions (17). Stringent quality filters (combined confidence scores ≥ 700, top 5% of distribution) were applied, yielding 265,834 high-confidence interactions among 17,869 unique proteins. Compound-protein interactions were obtained from STITCH database version 5.0 (18) and filtered identically, yielding 12,934 high-confidence compound-protein interactions.

#### 2.1.2 Flavonoid target and content data

Flavonoid data were obtained from two sources. First, flavonoid target proteins were identified through STITCH database by querying flavonoid structures for documented or predicted protein interactions. Second, quantitative flavonoid content in foods were obtained from USDA Database for the Flavonoid Content of Selected Foods Release 3.3, which provides mg/100 g measurements for 506 food products (19). Analysis focused on 14 flavonoids present in both databases: four flavonols (isorhamnetin, kaempferol, myricetin, quercetin), two flavones (apigenin, luteolin), three flavanones (eriodictyol, hesperetin, naringenin), and five flavan-3-ols [(-)-epicatechin, (-)-epicatechin-3-gallate, (-)-epigallocatechin, (-)-epigallocatechin-3-gallate, theaflavin].

#### 2.1.3 Pharmaceutical drug classification

FDA-approved drug information was obtained from PubChem through SPARQList, providing classification according to the Anatomical Therapeutic Chemical (ATC) system. The ATC system organizes medications hierarchically through five levels: Level 1 represents broad anatomical/therapeutic domains (14 categories A through V) and Level 2 represents therapeutic sub-groups within each domain. Analysis focused on ATC Levels 1 and 2, which provide clinically meaningful therapeutic categorization. Data for 3,739 FDA-approved drugs were retrieved, of which 1,496 drugs were integrated into the master network based on their presence in STITCH compound-protein interaction database.

#### 2.1.4 Data source justification

We used STRING/STITCH because they provide large, human-focused networks with unified confidence scores for proteins and small molecules, enabling stringent and consistent interaction filtering. The USDA flavonoid database and PubChem-ATC annotations were selected to translate compound-level networks into quantitative food profiles and clinically established therapeutic categories, respectively.

### 2.2 Master network construction

A master network was constructed by combining protein-protein and protein-compound interaction datasets filtered for combined scores ≥ 700 (top 5% of distribution). Conceptually, master network construction proceeded in four steps. First, we merged high-confidence STRING protein–protein interactions and STITCH compound–protein interactions after applying the combined score ≥ 700 filter. Second, we restricted compound nodes to the 14 dietary flavonoids with at least one STITCH-annotated human target and to 1,496 FDA-approved drugs present in STITCH. Third, we combined the protein and compound interaction layers into a single undirected network. Finally, we removed isolated nodes and duplicate edges and used the resulting graph as the master network for all downstream analyses. The integrated dataset contained 14 of 26 flavonoids from the USDA flavonoid database and 1,496 of 3,739 FDA-approved drugs; compounds lacking STITCH target data were excluded. Chemical compounds in the network were restricted to these 14 flavonoids and 1,496 drugs. Network construction and analysis were performed using Python version 3.10.9 with NetworkX version 3.1 for graph operations, NumPy version 1.23.5 and Pandas version 1.5.3 for data manipulation, and SciPy version 1.10.0 for statistical computations. The master network contained 19,379 nodes (17,869 proteins, 14 flavonoids, and 1,496 drugs) and 278,768 edges (265,834 protein-protein interactions and 12,934 protein-compound interactions).

### 2.3 Association analysis

#### 2.3.1 Network association strength of flavonoid and drug targets

To assess whether flavonoid target proteins were statistically enriched in therapeutic drug categories, Fisher's exact test based on the hypergeometric distribution was employed. For each flavonoid, drugs sharing common target proteins in the master network were identified, and their ATC classifications (Levels 1 and 2) were retrieved. The total number of drugs sharing target proteins with each flavonoid was calculated, along with the total number of drugs in each ATC category. Statistical significance was calculated using the hypergeometric probability:


$$\begin{array}{l} P=\overset{n}{\underset{i=x}{\sum }}\frac{\left(\begin{array}{c} M\\ i \end{array}\right)\left(\begin{array}{c} N-M\\ n-i \end{array}\right)}{\left(\begin{array}{c} N\\ n \end{array}\right)} \end{array}$$

where *N* represents the total number of drugs, M the number of drugs in a specific ATC category, *n* the number of drugs sharing target proteins with a given flavonoid, and x the number of drugs within n belonging to the specific ATC category. Associations with *p* < 0.05 were considered statistically significant. ATC association strength was presented as -log_10_ (*p*-value), providing a linear scale for downstream integration. This statistical enrichment approach differs from network proximity methods (16), which calculate shortest path distances between compound targets and disease proteins, providing a complementary perspective on polypharmacological mechanisms. To control for multiple testing, we applied the Benjamini-Hochberg false discovery rate (FDR) correction across all 1,330 statistical comparisons (14 flavonoids × 95 ATC categories spanning Levels 1 and 2). The procedure was implemented jointly across both ATC hierarchical levels to maintain family-wise error control. Although ATC Levels 1 and 2 form a hierarchical structure, we treated all tests as a single family because our primary goal was to control the overall proportion of false discoveries among all flavonoid–ATC associations, rather than to make separate inferences within each level. Both raw *p*-values and FDR-adjusted q-values are reported in Supplementary Table S1. Associations were considered statistically significant at FDR-adjusted *q* < 0.05, corresponding to a 5% expected false discovery rate among significant findings.

#### 2.3.2 Prediction of food–ATC therapeutic associations based on flavonoid profiles

For each of 506 foods in the USDA flavonoid database, a flavonoid content vector representing mg/100 g quantities of all 14 flavonoids was extracted. Of 81 ATC Level 2 categories, 32 categories showing statistically significant association strength (*p* < 0.05) with at least one flavonoid (from Section 2.3.1) were selected. Spearman rank correlation coefficients between each food's flavonoid profile and each ATC category's association strength vector were calculated (16,192 food-ATC pairs). This approach identifies foods whose flavonoid composition aligns with polypharmacological signatures associated with specific therapeutic categories. Food-ATC pairs ranking in the top 5% of correlation coefficients were selected as predicted therapeutic associations. This threshold balanced analytical stringency with feasibility of systematic downstream assessment across 286 candidate pairs; sensitivity analysis confirmed that the core set of food–ATC signatures remained stable across alternative thresholds (1%−10%). Hierarchical clustering of foods and ATC categories was performed using correlation distance with average linkage method.

### 2.4 Experimental validation: cell viability assays

#### 2.4.1 Cell culture

Jurkat human T leukemia cells were provided by Dr. T. Miyashita of the National Research Institute for Child Health and Development (Tokyo, Japan). The cell line was maintained in RPMI1640 medium supplemented with 10% fetal bovine serum, and 75 mg/L kanamycin sulfate. Cells were cultured at 37°C in a humidified atmosphere containing 5% CO_2_.

#### 2.4.2 Flavonoid treatment and MTT assay

Seven flavonoids were selected for experimental validation based on their network-predicted association strengths with antineoplastic drugs: high-association flavonoids (luteolin, myricetin, kaempferol, diosmetin) with *p*-values < 10^−7^, and low-association flavonoids (epicatechin, naringenin, hesperetin) with *p*-values > 0.05. Flavonoid stock solutions (10 mM in 100% dimethyl sulfoxide; DMSO) were serially diluted to generate eight logarithmically-spaced concentrations (1–200 μM). Jurkat cells (2 × 10^4^ cells/well) were seeded into 96-well plates and treated with flavonoid solutions (100 μL) to achieve final flavonoid concentrations. Diosmetin, the 4'-O-methyl ether of luteolin, was included as a structurally minimal variant of luteolin to probe the effect of B-ring methylation on both network-predicted association strength and experimental cytotoxicity. Control wells received vehicle (0.1% DMSO in RPMI1640). Following 23-h incubation, 10 μL of MTT solution (5 mg/mL in phosphate-buffered saline) was added to each well. After 1 h incubation, the supernatant was carefully removed and 100 μL of DMSO was added to dissolve formazan crystals. Absorbance at 570 nm was measured using a multi-well plate reader. Cell viability was calculated as the percentage of absorbance in treated wells relative to untreated vehicle control wells. All assays were performed as three independent biological experiments on separate days. In each experiment, four technical replicate wells per concentration were measured, and their mean absorbance was used as a single data point. Dose–response curves and LC_50_ values were calculated from the three biological replicates (*n* = 3). Error bars represent standard deviation across the three independent experiments.

### 2.5 Epidemiological literature assessment

To assess observational support for food-effect predictions, structured PubMed literature searches were conducted for each predicted food-effect relationship.

Search strategy and inclusion criteria: Search queries employed standardized format: [food name OR scientific name] AND [disease name OR therapeutic effect]. Study inclusion criteria required: (1) epidemiological study design with human subjects; (2) statistically significant associations (*p* < 0.05) between specific food and predicted health effect; (3) publication in English. Food names used in searches were consolidated categories (e.g., “grape,” “blueberry,” “garlic,” “tomato,” “black tea”) rather than specific formulations. This consolidation ensured that epidemiological evidence from diverse food preparations and forms (raw, cooked, processed, juice, frozen, canned) contributed to a unified assessment of each food source's health effects.

Screening and assessment: All searches and screening were conducted as of December 14, 2023. One reviewer performed the primary screening of titles and abstracts, and a second reviewer independently verified all potentially eligible studies; any discrepancies were resolved through discussion until consensus was reached. Disease names and therapeutic effect terms used for each ATC category, together with epidemiologically supported food-ATC associations, PMIDs, and full reference citations, are summarized in Supplementary Table S3.

#### 2.5.1 Food-level aggregation and category definition

Initial network pharmacology predictions were aggregated at the food category level rather than at the individual product level to enable equitable comparison across foods with inherent differences in product diversity. This approach addresses the biological principle that multiple product forms of a single food (e.g., fresh tomatoes, tomato juice, tomato sauce) often derive from identical plant sources and share overlapping phytochemical profiles. Five food categories with highest epidemiological support were defined: (1) Tomato products (6 forms), (2) Cranberry products (7 forms), (3) Tea products (4 varieties), (4) Orange products (4 forms), and (5) Blueberries (single form). Complete product lists are provided in Supplementary Table S3. Epidemiological evidence was summed within each category to enable comparison of cumulative research investment across foods.

#### 2.5.2 Analysis of unsupported high-confidence predictions

To systematically evaluate prediction accuracy and identify domain-specific research maturity biases, we extracted all food-ATC combinations ranking in the top 5% of Spearman correlation coefficients (ρ ≥ 0.609, *n* = 288) from the complete 16,192 food-ATC correlation matrix. These high-confidence predictions were cross-referenced against the epidemiological assessment results from Section 2.5. Predictions were classified as “epidemiologically supported” if supporting epidemiological evidence was identified through literature searches (*n* = 38, 13.2%), or “not yet supported” if no supporting evidence was found (*n* = 250, 86.8%).

For unsupported predictions, we quantified the distribution across ATC Level 2 categories and food items to identify systematic patterns. ATC categories and foods with the highest frequencies of unsupported predictions were identified and analyzed for potential mechanistic explanations, including consumption frequency, bioavailability constraints, and research maturity bias. Complete results are provided in Supplementary Table S4.

## 3 Results

### 3.1 Master network construction and flavonoid target diversity

**Figure 2:**
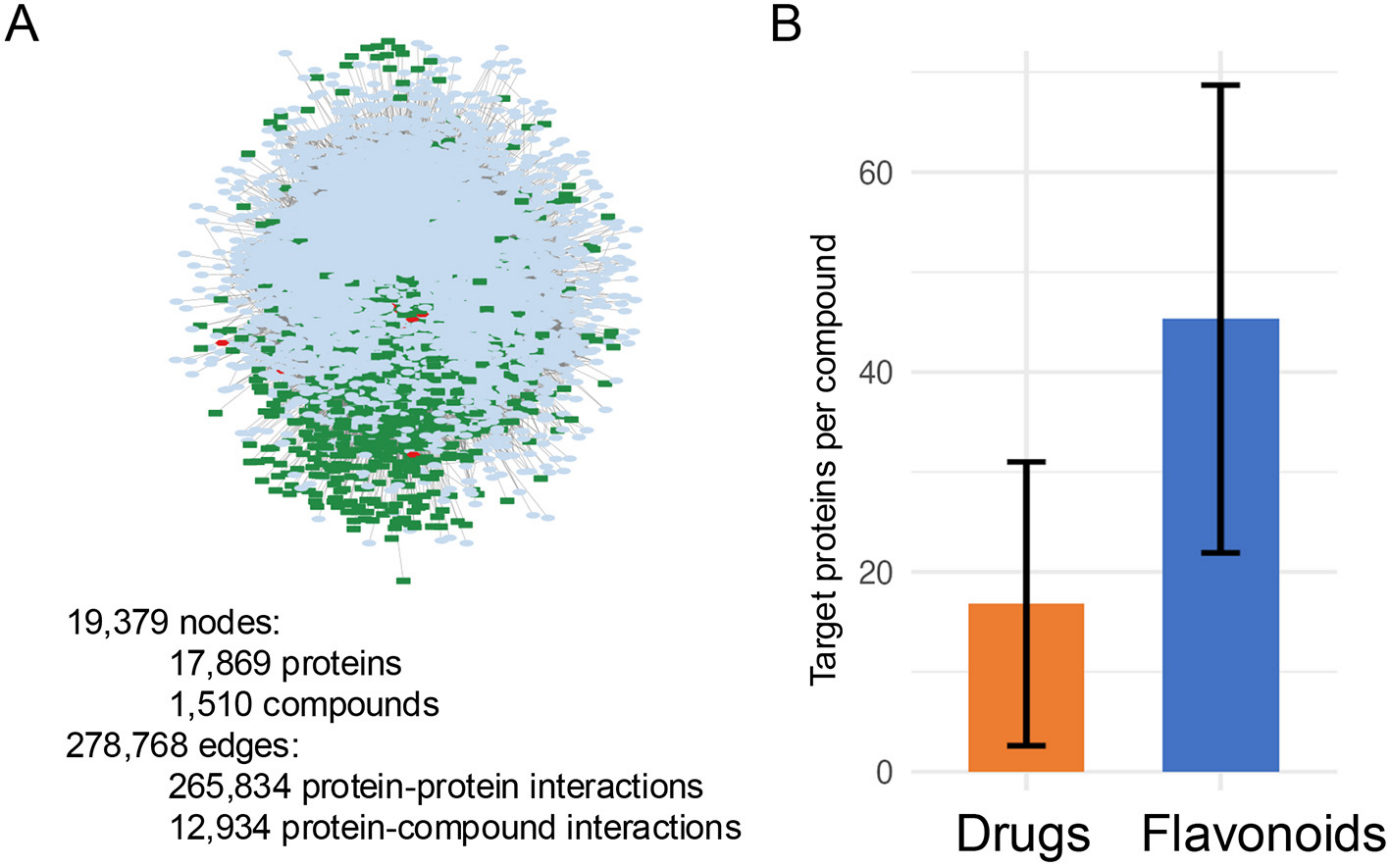
Master network architecture and target diversity. **(A)** Network visualization of 19,379 nodes and 278,768 interactions. Node types: human proteins (*n* = 17,869), flavonoids (*n* = 14), drugs (*n* = 1,496). Edges: protein-protein interactions (*n* = 265,834) and protein-compound interactions (*n* = 12,934). Scale-free topology (density 0.0015) reflects hub-and-spoke network architecture. **(B)** Target protein counts. Flavonoids (mean 45.3; range 16–98) exceed FDA-approved drugs (mean 16.8; range 1–187) by 2.7-fold (Wilcoxon *p* = 7.5 × 10^−4^).

The master network comprised 19,379 unique nodes representing either proteins or chemical compounds (Figure 2A). Among these nodes, 17,869 represented human proteins (95.8% of total nodes), while 1,510 represented chemical compounds (7.8% of total nodes), consisting of 14 distinct flavonoids and 1,496 FDA-approved drugs. These nodes were connected by a total of 278,768 unique interactions, of which 265,834 represented protein-protein interactions (95.4% of total edges) and 12,934 represented protein-compound interactions (4.6% of total edges). The network density was 0.0015 (0.15%), indicating a sparse network architecture typical of large-scale biological networks. The average node degree was 28.8 (SD 42.1). These topological metrics, as visualized in Figure 2A, characterize the master network as a large-scale, sparse, scale-free network with moderate modularity, reflecting the natural organization of biological interaction systems.

When analyzing target protein diversity, flavonoids and FDA-approved drugs displayed striking differences (Figure 2B). The 14 flavonoids targeted a mean of 45.3 proteins per compound (range 16–98), with myricetin (98 targets), kaempferol (87), quercetin (76), and luteolin (68) showing the highest promiscuity. In contrast, 1,496 FDA-approved drugs targeted a mean of 16.8 proteins per drug (range 1–187), with 243 drugs (16.2%) following the classical “magic bullet” paradigm of 1–2 targets (6). This 2.7-fold difference was statistically significant (Wilcoxon rank-sum test: Z = 3.78, *p* = 7.5 × 10^−4^), confirming that flavonoids' multi-target profiles substantially exceed those of conventional pharmaceuticals.

### 3.2 Flavonoid-drug category association analysis

We quantified flavonoid-ATC associations using Fisher's exact test, with association strength defined as –log_10_ (*P*-value), providing a linear scale for statistical significance. Higher values indicate stronger associations (*p* < 0.05 corresponds to association strength > 1.3). Surprisingly, only 42 of 252 possible flavonoid-disease combinations (16.7%) showed statistical significance, despite strong epidemiological evidence linking flavonoid consumption to disease prevention. To determine whether this limited direct association was specific to flavonoids, we conducted parallel analysis for FDA-approved drugs across the same disease categories. Drug-disease gene associations were similarly constrained: only 27.7% of drugs demonstrated statistically significant associations (*p* < 0.05) with at least one disease category, despite known therapeutic efficacy. This parallel finding suggested that both natural products and synthetic pharmaceuticals achieve therapeutic effects predominantly through indirect network mechanisms rather than direct targeting of disease genes (20), consistent with recent findings that many drugs act through pathway-level perturbations.

Given these constraints of direct disease association approaches, we reoriented our analysis toward mechanism-centric examination. We investigated statistical associations between flavonoid target proteins and therapeutic drug categories to identify potential flavonoid pharmacological mechanisms based on overlaps with established drug targets. Supplementary Table S1 presents complete *p*-value matrices quantifying associations between all 14 flavonoids and ATC categories at both Level 1 (14 broad therapeutic domains) and Level 2 (77 specific therapeutic subcategories), with *p* < 0.05 indicating statistical significance.

**Figure 3:**
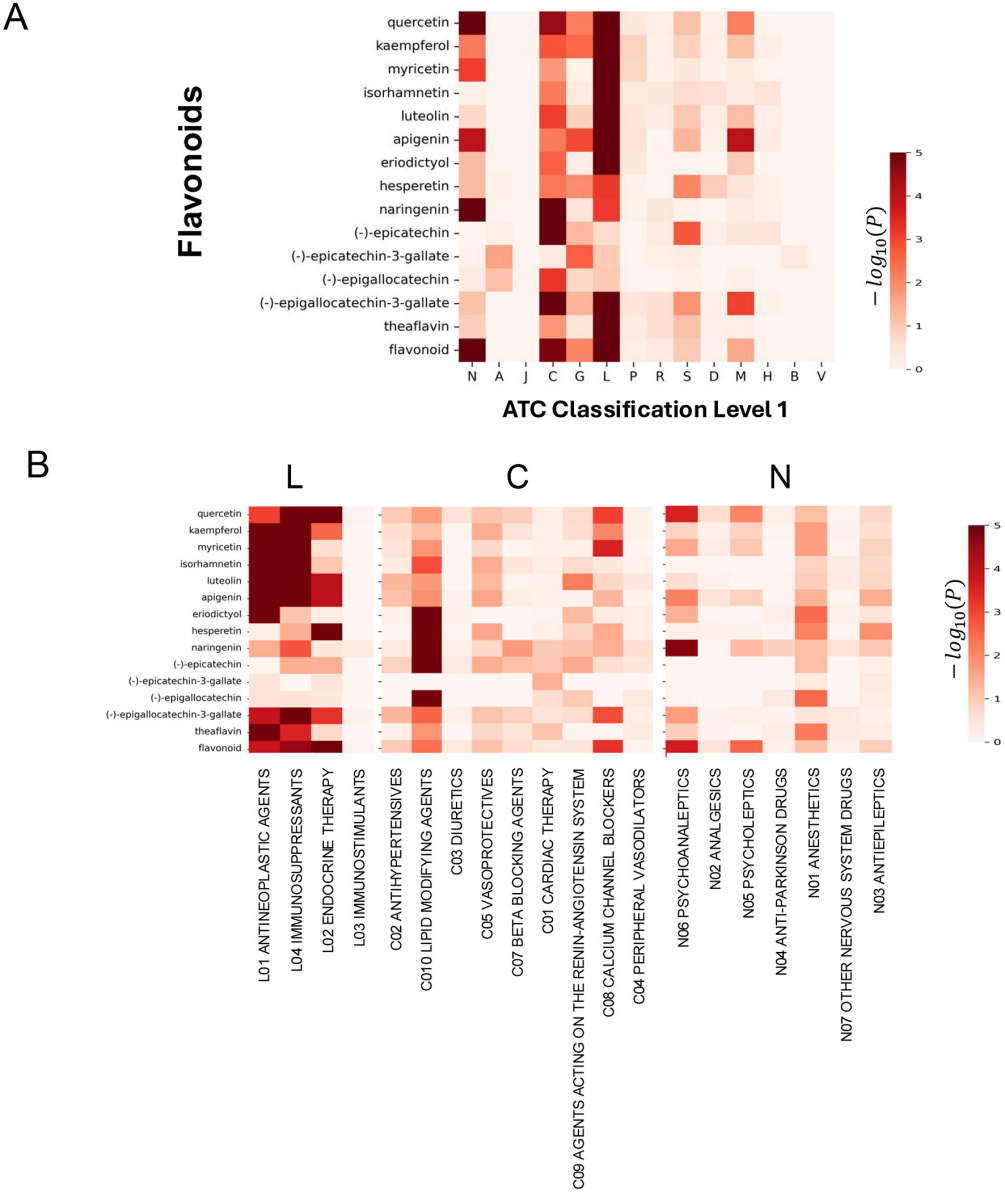
Flavonoid-ATC association analysis. Heatmaps quantifying statistical association strength between 14 flavonoids and therapeutic drug categories via Fisher's exact test. **(A)** ATC Level 1 analysis across 14 broad therapeutic domains (–log_10_ *p*-values). **(B)** ATC Level 2 analysis across 20 specific therapeutic categories from ATC Level 1 categories L, C, and N.

Investigation of these associations revealed extensive overlap at the first level of ATC classification (Figure 3A). Ten of the 14 flavonoids (71.4%) showed significant associations with cardiovascular drugs (ATC-C), reflecting established epidemiological evidence. More striking, eleven of the 14 flavonoids (78.6%) showed associations with antineoplastic and immunomodulatory agents (ATC-L), and four of the 14 (28.6%) with nervous system agents (ATC-N). These high proportions demonstrate that flavonoid target proteins align substantially with therapeutic drug targets across multiple therapeutic domains.

Deeper analysis at the second level of ATC classification revealed particularly striking associations with antineoplastic agents (ATC L01), as shown in Figure 3B. Luteolin emerged as the most strongly associated flavonoid, with an association *p*-value of 2.94 × 10^−11^, corresponding to a –log_10_*P*-value of 10.53. Theaflavin followed closely with *p* = 2.9 × 10^−11^ (–log_10_*P* = 10.54), myricetin demonstrated *p* = 1.29 × 10^−10^ (–log_10_*P* = 9.89), isorhamnetin showed *p* = 8.60 × 10^−10^ (–log_10_*P* = 9.01), and kaempferol exhibited *p* = 5.69 × 10^−7^ (–log_10_*P* = 6.24). Beyond these five top-tier associations, seven additional flavonoids showed moderately strong associations with antineoplastic agents at *p* < 10^−3^. Collectively, 11 of the 14 flavonoids (78.6%) demonstrated statistically significant associations with antineoplastic drug targets at *p* < 0.05, indicating near-universal overlap in antineoplastic-relevant target pathways.

### 3.3 Experimental validation: quantitative correlation between network prediction and bioactivity

**Figure 4:**
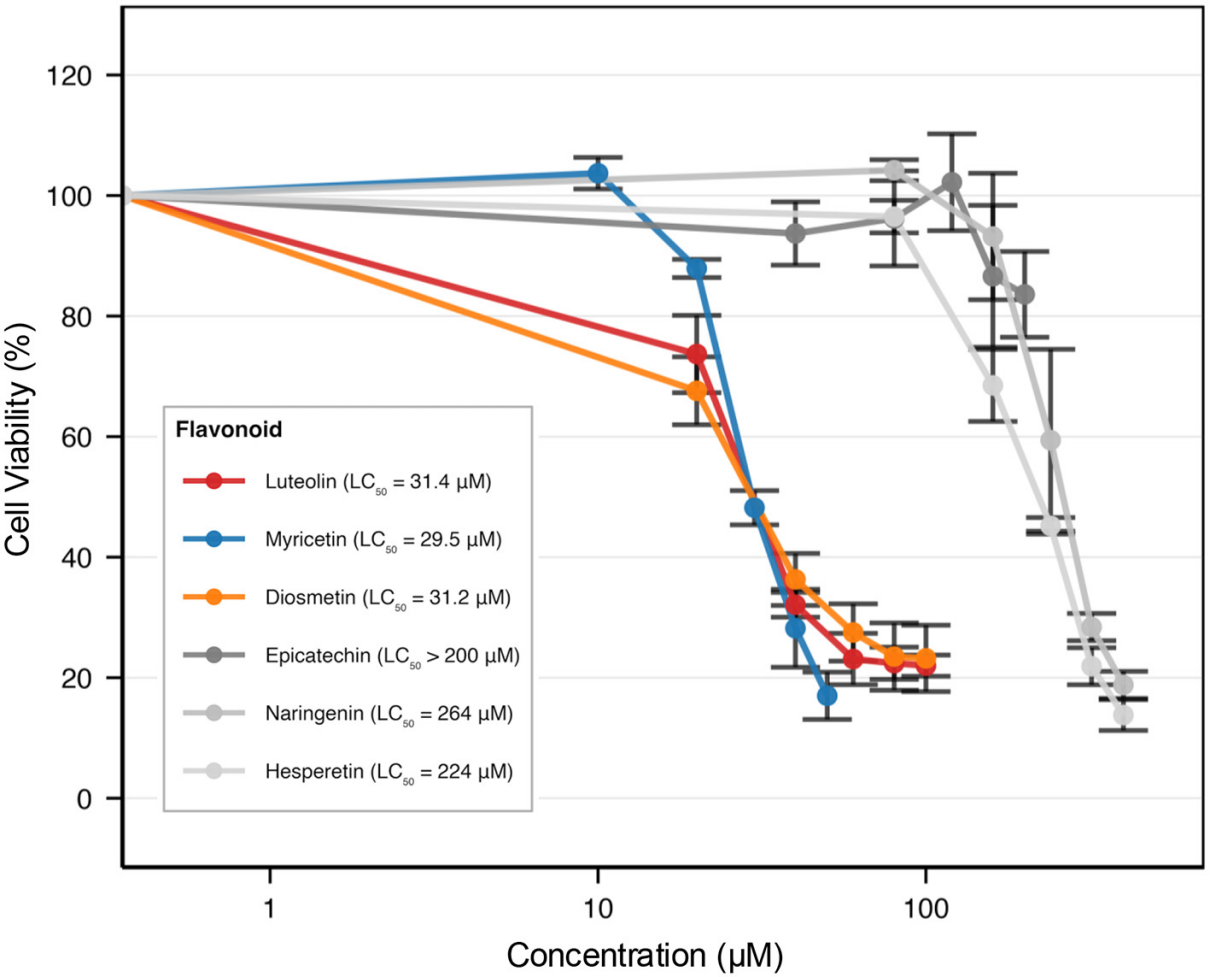
Dose-response cytotoxicity curves. Dose-response curves (1–200 μM) for seven flavonoids in Jurkat leukemia cells. Orange lines: high-association flavonoids (luteolin, myricetin, diosmetin). Gray lines: low-association flavonoids (epicatechin, naringenin, hesperetin). Cell viability expressed as percentage of vehicle control (mean±SD, *n* = 3 independent experiments, each with 4 technical replicates) determined via MTT assay.

To test whether network-predicted association strengths translate into observable cellular bioactivity, we conducted dose-response cell viability assays using Jurkat leukemia cells. As shown in Figure 4, flavonoids with high association strength for the antineoplastic category (*p*-values < 10^−7^) demonstrated substantial cytotoxic activity. Luteolin exhibited an LC_50_ of 31.4 μM, and myricetin demonstrated 29.5 μM. In striking contrast, flavonoids with low association strength (*p*-values > 0.05) showed substantially reduced potency, with LC_50_ values exceeding 200 μM for epicatechin, naringenin, and hesperetin.

**Figure 5:**
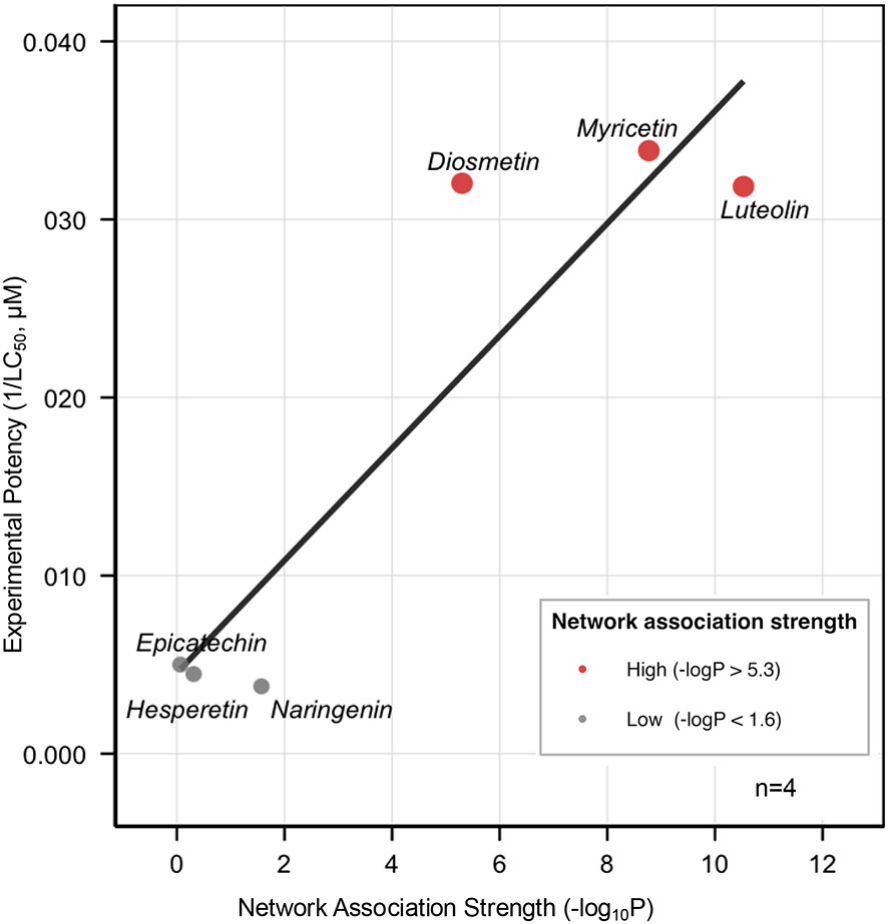
Network prediction vs. experimental bioactivity correlation. Correlation between network-predicted association strength (–log10 *p*-value) and experimental potency (1/LC_50_, μM^−1^). High-association flavonoids (red circles: luteolin, myricetin, diosmetin) show strong cytotoxicity; low-association flavonoids (gray circles: epicatechin, hesperetin, naringenin) show minimal activity. Linear regression (Pearson *r* = 0.918; *p* = 0.0098; R^2^ = 0.843) demonstrates that network predictions explain 84.3% of bioactivity variance.

Correlation analysis revealed strong concordance between network association strengths and experimental bioactivity (Figure 5). Pearson correlation comparing network association strength (–log_10_*P* values) with experimental potency (–log_10_ LC_50_) yielded *r* = 0.918 (*p* = 0.0098; R^2^ = 0.843), indicating that network predictions explain 84.3% of variance in observed bioactivity.

Independent validation tested whether this correlation generalizes beyond the initial 14 flavonoids. Network analysis of diosmetin, included in the master network but not used for food-level predictions, predicted an antineoplastic association strength (–log_10_*P* = 5.32). Experimental measurement yielded an LC_50_ of 31 μM, demonstrating close agreement with the predicted range and confirming that the predictive framework generalizes to novel compounds (Figure 5).

### 3.4 Food-level therapeutic effect predictions

**Figure 6:**
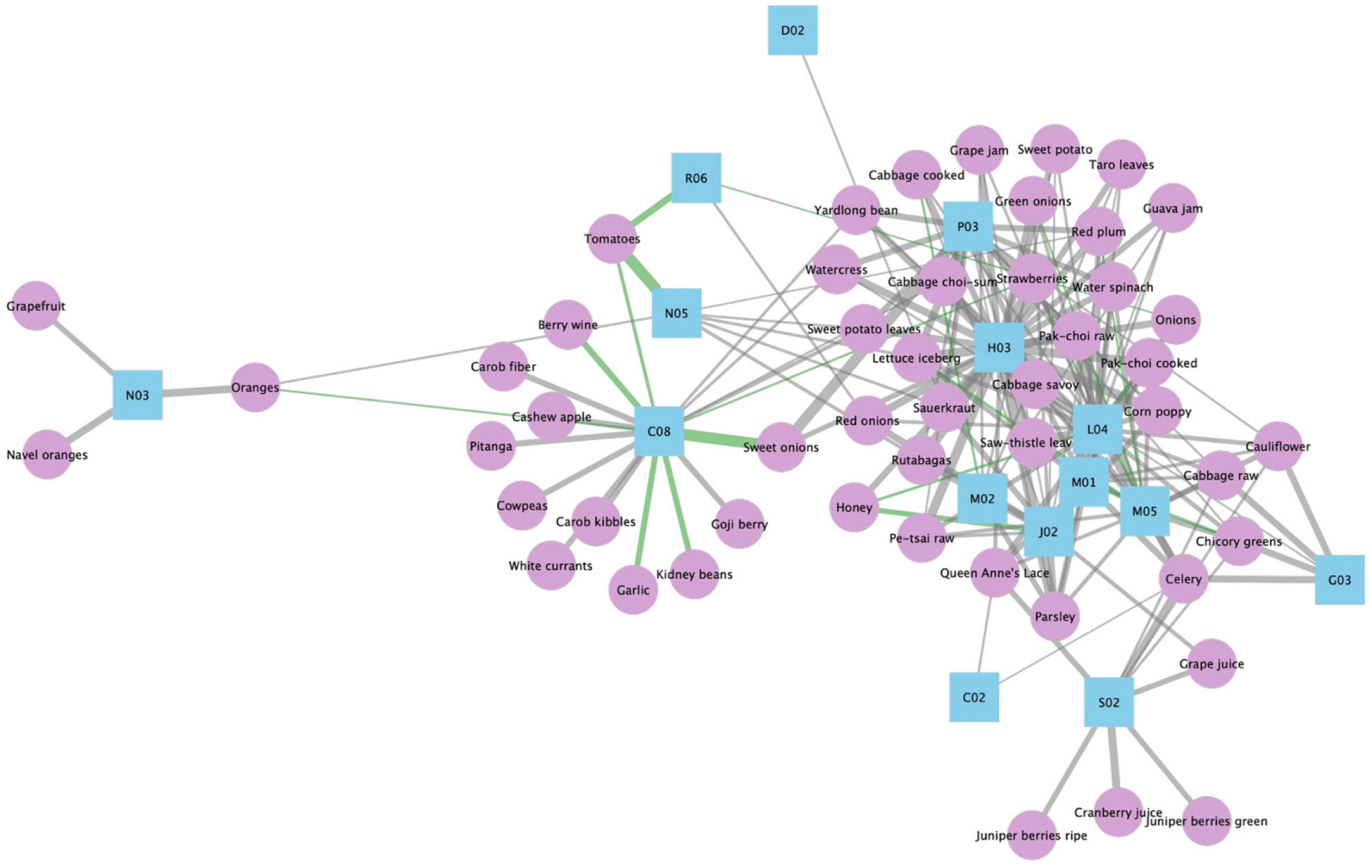
Epidemiologically supported and predicted food–ATC therapeutic network. Network showing associations between flavonoid-rich foods (purple nodes, *n* = 50) and ATC therapeutic categories (blue nodes, *n* = 15) based on top 5% Spearman correlation strength (ρ ≥ 0.60). Green edges (*n* = 19, 10.9%) represent associations supported by epidemiological evidence (detailed in Section 3.5 and Supplementary Table S3); gray edges (*n* = 155, 89.1%) represent computationally predicted associations. Edge width is proportional to correlation coefficient. Node size for ATC categories reflects degree centrality (number of connected foods).

To translate network associations into dietary predictions, we integrated USDA flavonoid content data with association strengths for 32 ATC Level 2 categories that demonstrated statistically significant flavonoid associations. For each of 506 foods, flavonoid profiles were compared against therapeutic category vectors using Spearman rank correlation (16,192 food-ATC pairs). Positive correlations (ρ > 0) were classified as predicted therapeutic effects, yielding 685 food-ATC associations across 216 distinct foods and 26 ATC categories. Among these 685 predictions, food-ATC pairs ranking in the top 5% of correlation coefficients (ρ ≥ 0.60) were classified as high-confidence associations, yielding 174 high-confidence associations across 50 distinct foods and 15 ATC categories (Figure 6). Subsequent epidemiological literature assessment (detailed in Section 3.5) identified published support for 19 of these 174 high-confidence associations (10.9%), shown as green edges in Figure 6. The complete 218 × 26 correlation matrix is provided in Supplementary Table S2.

Among the predicted associations, tea varieties showed particularly strong correlations with C01 (cardiac therapy; ρ = 0.547–0.603), contrasting with the majority of foods that showed negative correlations (mean = −0.24, SD = 0.238). Tea consistently ranked in the top positions for C01 associations, suggesting that tea's flavonoid profile aligns with cardiac therapy drug targets.

### 3.5 Epidemiological literature assessment

To assess whether network-predicted food-effect relationships correspond to documented health associations, systematic PubMed literature searches were conducted for each of the 685 predicted food-ATC category associations (Supplementary Table S2). Our literature searches identified 241 pieces of epidemiological supporting evidence, identifying epidemiological support for 96 food-ATC category associations (14.0% overall epidemiological support rate). Among these 96 epidemiologically supported associations, 19 involved high-confidence predictions (ρ ≥ 0.609), shown as green edges in Figure 6. These 96 epidemiologically supported predictions were collectively supported by 132 unique references, yielding an average of 2.5 evidence pieces per supported prediction (range: 1–9 studies per prediction) (Supplementary Table S3).

**Figure 7:**
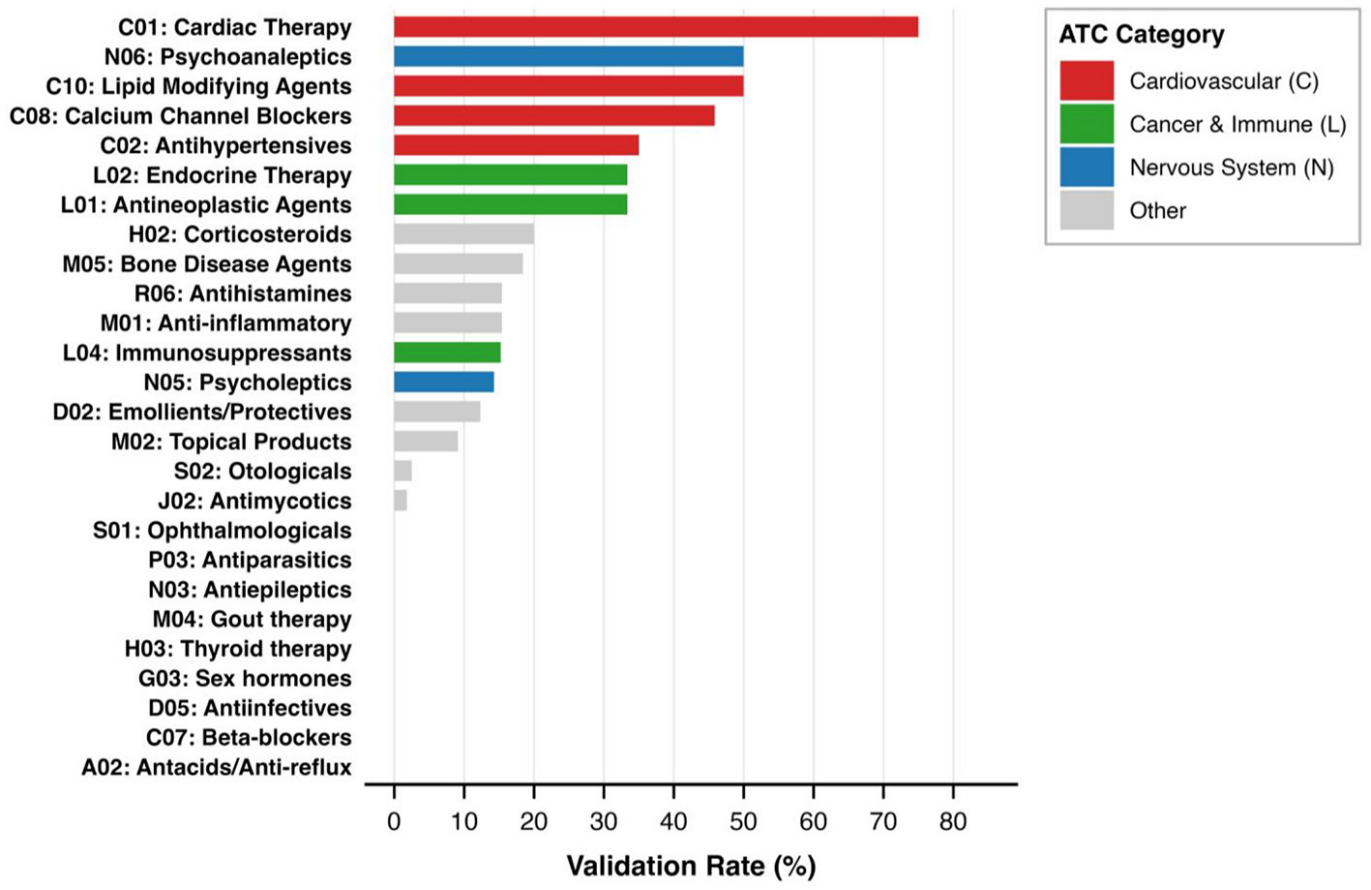
Epidemiological support rates by therapeutic domain. Bars represent support rates (%) for food-ATC associations across 26 therapeutic categories, color-coded by domain: cardiovascular (C, dark red), cancer/immune (L, medium red), nervous system (N, light red), and other (gray). Cardiovascular categories achieved highest rates (47.1% combined).

Epidemiological support rates varied substantially across therapeutic domains, as shown in Figure 7. Cardiovascular categories demonstrated the highest rates: cardiac therapy 75% (6/8 predictions), lipid-modifying agents 50% (2/4), calcium channel blockers 45.8% (33/72), antihypertensives 35.0% (7/20), and antineoplastic agents 33.3% (1/3). The combined cardiovascular categories achieved 47.1% (48/102). Intermediate rates appeared in endocrine therapy (33.3%, 2/6), corticosteroids (20.0%, 1/5), bone disease agents (18.4%, 7/38), psycholeptics (14.3%, 9/63), anti-inflammatory agents (15.4%, 6/39), antihistamines (15.4%, 2/13), immunosuppressants (15.2%, 7/46), and emollients (12.3%, 9/73). Lower rates were observed in topical pain products (9.1%, 1/11), otologicals (2.5%, 1/40), and antimycotics (1.8%, 1/56). When aggregating all related products within each food category to enable consistent cross-category comparison, five food categories emerged with the highest epidemiological support evidence:

Tomato products demonstrated the strongest cumulative epidemiological support with 40 evidence counts distributed across six products (fresh tomatoes: 13 counts; tomato juice: 8 counts; catsup, marinara sauce, tomato paste, tomato purée: combined 19 counts). These literature-supported associations spanned four therapeutic domains: cardiovascular protection (C08), psycholeptic agents (N05), psychoanaleptic agents (N06), and antihistamine agents (R06).

Cranberry products ranked second with 33 evidence counts across seven distinct products including dried cranberries, cranberry juice cocktail, and cranberry sauce (each category: 7 counts) plus four additional cranberry formulations. Cranberry literature-supported associations clustered in cardiac therapy (C02), cardiovascular agents (C08), immunosuppressants (L04), and anti-inflammatory agents (M01), reflecting its traditional use in urinary tract and cardiovascular health.

Tea products ranked third with 30 evidence counts concentrated exclusively in cardiac therapy (C01). These literature-supported associations derived from four distinct tea varieties: black tea (9 studies), decaffeinated green tea (7 studies), flavored green tea (7 studies), and Quingmao green tea (7 studies), demonstrating consistent therapeutic specialization in vasodilation, blood pressure regulation, and arrhythmia prevention.

Orange products accumulated 20 evidence counts across four product forms (fresh oranges, orange juice, sour orange jam) and four therapeutic categories (C08, C10, N03, N05).

**Figure 8:**
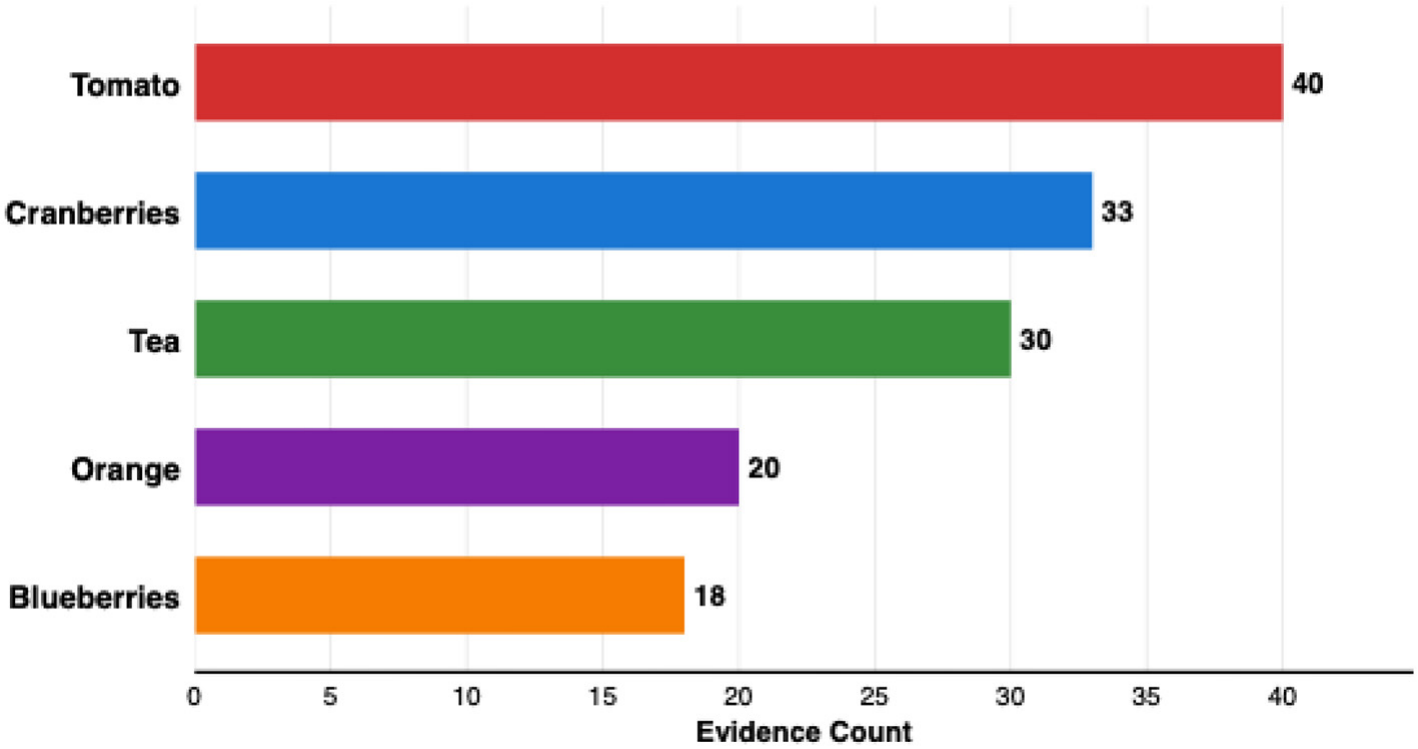
Top five food categories by epidemiological support. Counts represent cumulative evidence across all products within each food category (e.g., Tomato includes fresh tomatoes, juice, paste, and sauce; Tea includes black, green decaffeinated, and flavored varieties).

Blueberries represented a unique epidemiological support pattern: despite a single product form (frozen, unsweetened), it achieved 18 evidence counts and demonstrated the broadest therapeutic reach spanning six ATC categories (C02, C08, L04, M01, M05, N05), suggesting diverse bioactive mechanisms (Figure 8).

### 3.6 Unsupported predictions and research maturity bias

To evaluate prediction coverage and identify systematic gaps in epidemiological support, we analyzed the 288 food-ATC combinations ranking in the top 5% of correlation scores (ρ ≥ 0.609). Among these high-confidence predictions, 38 (13.2%) were epidemiologically supported, while 250 (86.8%) remained not yet supported (Supplementary Table S4).

Unsupported predictions showed distinct patterns across both therapeutic categories and food types. ATC categories D02 (dermatologicals, 42 unsupported predictions), H03 (thyroid therapy, 39), L04 (immunosuppressants, 29), and P03 (ectoparasiticides, 28) accounted for 55.2% of predictions lacking epidemiological support. Foods with the highest numbers of unsupported predictions included parsley (*n* = 7 predictions), cruciferous vegetables (*n* = 6), and celery (*n* = 6).

## 4 Discussion

### 4.1 Multi-evidence support for network pharmacology predictions

The multi-evidence framework established here integrates network pharmacology with experimental and observational evidence as a systematic approach for characterizing dietary polypharmacological properties. The convergence of computational predictions, experimental validation (*r* = 0.918, R^2^ = 0.843), and epidemiological consistency (47% epidemiological support in cardiovascular domains) demonstrates that integrating multiple independent evidence streams can reliably predict flavonoid bioactivity and identify food-therapeutic associations.

Flavonoids exhibit 2.7-fold greater target diversity than conventional drugs (45.3 vs. 16.8 proteins, *p* = 7.5 × 10^−5^), reflecting fundamental differences in molecular design between natural products and rationally designed pharmaceuticals (7, 21). The strong correlation between network-predicted association strengths and experimental bioactivity (*r* = 0.918, R^2^ = 0.843) confirms that network predictions reliably capture flavonoid bioactivity, supporting the framework's predictive foundation.

While our validation employed Jurkat T-cell leukemia cells as a single model system, published data demonstrate that the rank order of flavonoid potency observed in our experiments generalizes across diverse cancer types. Luteolin, which showed the strongest predicted antineoplastic association (log *p*-value = 10.53) and highest experimental potency (LC_50_ = 31.4 μM in Jurkat cells), exhibits comparable cytotoxicity in HepG2 hepatocellular carcinoma [IC50 = 31.4 μM; (22)], MCF-7 breast adenocarcinoma [IC50=41.9 μM; (23)], and HCT116 colorectal carcinoma [30 μM induces growth inhibition; (24)]. Similarly, myricetin demonstrates moderate potency across multiple cell lines: A2780/OVCAR3 ovarian cancer [IC50=25 μM; (25)], HepG2 hepatoma [IC50=87.8 μM; (26)], and MCF-7 breast cancer (27). In contrast, epicatechin remains inactive at physiologically relevant concentrations across all models [IC50 >100 μM; (28)]. Experimental support in this study is limited to *in vitro* cytotoxicity in Jurkat T-cell leukemia cells and should not be generalized to other therapeutic domains without additional testing. Nevertheless, this consistent rank order across cell types supports the framework's predictive relevance beyond a single cell line.

Our results parallel Valle et al.'s (16) demonstration that network proximity between polyphenol targets and disease proteins predicts therapeutic effects, as validated by rosmarinic acid's platelet inhibition. While their study employed network proximity metrics based on shortest path distances, our statistical enrichment approach identifies shared pharmacological neighborhoods between flavonoids and therapeutic drug categories, offering complementary mechanistic insights into polypharmacological effects of dietary bioactives.

### 4.2 Flavonoid-drug category associations reveal network-mediated therapeutic mechanisms

A striking parallel emerged from our analysis: flavonoids and FDA-approved drugs show strikingly low direct associations with disease-related genes (flavonoids: 16.7%, drugs: 27.7% of tested combinations), yet both achieve therapeutic effects through indirect network mechanisms. This systematic dissociation reveals a fundamental principle: both natural products and synthetic pharmaceuticals achieve therapeutic efficacy not through direct targeting of disease genes, but through coordinated multi-target perturbations of interconnected biological networks. The convergence between flavonoids and drugs at the therapeutic mechanism level—despite low direct disease-gene associations—supports a network-mediated model of drug action that transcends the traditional “magic bullet” paradigm. Complex diseases likely benefit from coordinated multi-target perturbations rather than single-target selectivity (15, 20), consistent with emerging polypharmacological approaches to drug discovery.

The broad alignment of flavonoid targets with cardiovascular drug mechanisms provides computational support for epidemiological benefits observed in flavonoid-rich diets (4, 29, 30). This alignment demonstrates the framework's biological relevance. Critically, the near-universal association between flavonoids and antineoplastic agents (78.6% of flavonoids; 11 of 14 compounds) indicates a class-wide therapeutic signature rather than isolated bioactivity. This pattern indicates that flavonoid target profiles align with therapeutic networks engaged by established anticancer drugs, suggesting that dietary flavonoid combinations may exert coordinated polypharmacological effects. Furthermore, the significant engagement of 28.6% of flavonoids with nervous system drug targets highlights under-explored neuroactive mechanisms, suggesting computationally inferred potential for dietary strategies in neurological health beyond established cardiovascular and metabolic domains.

### 4.3 Epidemiological support, false positives, and research maturity bias

The 14.0% overall epidemiological support rate is more appropriately interpreted as reflecting uneven research maturity across therapeutic domains rather than intrinsic limitations of the framework. Multi-level analysis provides complementary perspectives: 31.6% of foods demonstrated at least one supported prediction, and 64.0% of therapeutic categories received epidemiological support, indicating that the framework preferentially recapitulates relationships in well-studied cardiovascular and metabolic domains while generating hypotheses in understudied areas. Cardiovascular domains achieved the highest epidemiological support rates (47.1% overall, with cardiac therapy reaching 75%), consistent with the extensive cardiovascular literature documenting flavonoid-rich foods as cardioprotective (4, 29, 30). In contrast, domains such as psycholeptics (14.3%), otologicals (2.5%), and antimycotics (1.8%) showed substantially lower support rates, reflecting gaps in epidemiological research priorities. Among unsupported high-confidence predictions, systematic patterns emerged: dermatologicals (D02), thyroid therapy (H03), immunosuppressants (L04), and ectoparasiticides (P03) accounted for over half of unsupported predictions, despite extensive *in vitro* literature documenting flavonoid bioactivity in these domains. Similarly, foods consumed in small quantities (parsley, celery, cruciferous vegetables) showed high predicted associations yet lack epidemiological support. For example, cruciferous vegetables showed predicted thyroid drug associations consistent with known goitrogen effects (31), but typical consumption levels have not been evaluated in controlled trials. Bioavailability variability mediated by gut microbiota (32) and incomplete translation of protein-level target overlap to clinical efficacy likely contribute to these gaps.

These unsupported predictions represent testable hypotheses that may guide future clinical trials and observational studies in therapeutic domains where diet-disease relationships remain underexplored (13). As nutritional epidemiology expands beyond traditional cardiovascular and metabolic research, the framework's predictive accuracy in emerging domains can be systematically evaluated.

### 4.4 Toward network-informed precision nutrition

Food-category aggregation revealed that polypharmacological signatures—quantified through network-informed and epidemiologically supported food-ATC associations—differentiate foods along a spectrum from broad-spectrum modulators (tomato: 40 literature-supported associations across 4 categories; blueberry: 6 ATC categories) to domain-specific interventions (tea: cardiac therapy exclusively). Rather than establishing causality, this differentiation provides a conceptual framework for hypothesis-driven precision nutrition, in which food bioactivity profiles inform the generation of testable strategies tailored to individual health needs.

Critically, this framework operates at the food level rather than isolating single nutrients, aligning with whole-food dietary paradigms. The convergence of computational predictions, experimental assays, and epidemiological consistency demonstrates that our network pharmacology can systematically quantify multi-target properties of dietary bioactives. However, current evidence from observational studies reflects literature availability rather than mechanistic proof or causal confirmation. Translating these signatures into clinical recommendations requires controlled intervention trials that establish causality and dose-response relationships for specific health outcomes.

### 4.5 Limitations and future directions

Our analysis has several constraints. We examined only 14 of the 26 flavonoids in the USDA database because the remaining compounds lacked sufficient STITCH target coverage, which may bias the network toward well-characterized flavonoids. Moreover, low oral bioavailability and unmodeled inter-individual differences in flavonoid metabolism and gut microbiota complicate direct translation of *in vitro* potency to *in vivo* effects. Quercetin absorption from onions (~52%) substantially exceeds that from tea (~17%) despite similar content, reflecting glycoside-dependent bioavailability differences not captured in content-based predictions (32). Integration of pharmacokinetic parameters from human studies would improve prediction accuracy.

A fundamental limitation is that foods contain diverse bioactive compounds beyond the 14 flavonoids analyzed here, whose contributions to observed health effects remain unresolved. Tomatoes, for example, contain lycopene (2.5–3.7 mg/100g in fresh fruit) at concentrations exceeding total flavonoid content, and lycopene's cardiovascular protective effects are well-established (33). Similarly, blueberries and cranberries contain high concentrations of anthocyanins (>250 mg/100 g) and proanthocyanidins, respectively—both flavonoid subclasses excluded from our 14-compound analysis due to limited target data —with documented cardiovascular and antibacterial activities. The framework's focus on 14 flavonoids necessarily overlooks synergistic or antagonistic interactions among co-occurring bioactives, potentially underestimating or overestimating true therapeutic potential.

Despite this limitation, the framework successfully predicted food–ATC therapeutic signatures based on 14 flavonoids alone, achieving 14.0% of predictions receiving epidemiological support overall and 47.1% support in cardiovascular domains. This demonstrates that focused analyses of specific bioactive classes can capture meaningful pharmacological signals even though the observed food-level associations may also reflect contributions from other, unmodeled bioactive compounds.

Future priorities include pharmacokinetic modeling, pathway-level validation, and integration of additional bioactive classes to enable comprehensive food profiling. The framework demonstrated that network pharmacology can systematically predict food-related therapeutic signatures using compositional data alone and that these signatures can be supported by *in vitro* assays and observational evidence. Together, these findings establish a computational foundation for generating testable hypotheses for precision nutrition and for informing evidence-based dietary guidance.

## Data Availability

The original contributions presented in the study are included in the article/Supplementary material, further inquiries can be directed to the corresponding author.
